# The Characterization of Normal Male and Female Voice from Surface Electromyographic Parameters

**DOI:** 10.3390/jpm14060592

**Published:** 2024-06-01

**Authors:** Clara Puig-Herreros, José Luis Sanz, Luz Barona-Lleó, Leopoldo Forner, Vicent Rosell-Clari

**Affiliations:** 1 Speech Therapy University Clinic, Department of Basic Psychology, Universitat de València, Avda. de Blasco Ibáñez, 21, 46010 Valencia, Spain; 2 Department of Stomatology, Faculty of Medicine and Dentistry, Universitat de València, C/Gascó Oliag 1, 46010 Valencia, Spain; 3 Department of Otolaryngology, Barona Clinic, “Casa de Salud” Hospital, Calle Manuel Candela, 41, 46021 Valencia, Spain

**Keywords:** voice, characterization, surface electromyography

## Abstract

Currently, there is no consensus on the characterization of the human voice. The objective of the present study is to describe the myoelectric behavior of the extrinsic musculature of the larynx in 146 people with normal voice (Spanish speakers), aged between 20 and 50 years old. Different vocal tasks were recorded using a surface electromyograph (SEMG). In all vocal tasks, it was observed that women had higher activation (µV) in the suprahyoid and sternocleidomastoid muscles than men, while men had higher activation in the infrahyoid muscles. SEMG is a valid procedure to help define normal vocal characteristics in the studied population, providing reference values during clinical examination. However, it is necessary to adopt a universal system of assessment tasks and standardized measurement techniques to allow for comparisons with future studies.

## 1. Introduction

Among the procedures for the study of muscle electrical activity are electromyography (EMG) and surface electromyography (SEMG). The former analyzes and records the myoelectric activity of muscle fibers for the diagnosis of neuromuscular diseases, using needle insertion [1]. SEMG uses surface electrodes, one of which serves as a reference (neutral electrode) located in the vicinity of electrically inactive tissue such as bones or tendons, for example, in our case, as will be described below, in the olecranon, in order to avoid interference.

Surface electromyography (SEMG) records myoelectric activity (depolarization of the muscle cell membrane [2]) and contributes to the diagnosis of motor disorders [3] and, specifically, that of the most superficial muscles, identifying their contribution (and the importance of the same) in a given task [4]. This technique is based on the detection of the action potential of muscle fibers and motor units in the surrounding tissues or the skin during muscle activity [5]. The signal is represented through voltage, measured in µV. SEMG allows researchers to know the muscle excitation and describe the muscle patterns, giving information about the recruited motor units in a non-invasive way [6]. 

SEMG is used in a wide variety of disciplines, such as physiology, engineering, biomechanics, medical sciences, and clinical neuroscience [7,8], but in speech therapy, the use of SEMG is not very frequent. Speech–language pathologists and speech therapists use it to help in the evaluation, diagnosis, and therapy of different functions, especially stomatognathic functions such as chewing and swallowing, proper to the orofacial motor area [9], as well as the masticatory muscles’ activity [10]. It is a non-invasive technique, easy to apply, and reports muscle activity in real time [11].

The electromyogram obtained is the result of the sum of the motor unit action potentials produced during the contraction of the neuromuscular units [7,12,13]. The amplitude (maximum height of the action potential) is expressed in microvolts, while the frequency is measured in hertz, indicating the number of times the potential is repeated per unit of time (usually in one second) [5].

Recently, high-density surface electromyography [14] has been used to study muscles associated with swallowing. This technique appears to be useful for the analysis of the motor unit recruitment in the suprahyoid muscles—mylohyoid, geniohyoid, digastric, and stylohyoid [15]. 

The standardization of the recording, analysis, and interpretation of the electromyographic signal is a pending issue and urgently needed, for which interesting projects have been initiated [16].

Research related to the study of the voice together with SEMG is scarce and with a great dispersion in the aspects analyzed, from the sample size to the vocal tasks that have been developed or the muscles studied [3].

The aim of this cross-sectional study is to analyze the most relevant surface electromyographic parameters during vocal emission in Spanish speakers, in a sample of 146 adults (women and men), for the characterization of normality patterns of muscular activity in vocal production tasks. 

The hypotheses proposed are as follows: (1) there is a characteristic pattern of SEMG signals associated with normal voice emission, without vocal pathology, and (2) there are differences between sexes.

## 2. Materials and Methods

### 2.1. Study Design

Taking into account the objectives of this study, a cross-sectional descriptive design was chosen. Before starting the study, a favorable report on the project was obtained from the Human Research Ethics Committee (CEIH) of Universitat de València with procedure number 1054131.

### 2.2. Participants

The sample consisted of a total of 146 people (after calculating the sample size), all Spanish speakers, comprising 72 males and 74 females (self-identified as such) between 20 and 50 years of age. All participants who presented a subjective perception of normal voice (both by the person participating in the study and by 3 members of the research team, all voice specialists) were included, as well as those without known vocal pathology (both organic and functional) at the time of the examination and without relevant medical history.

### 2.3. Materials

The MioTool Face surface electromyograph from Miotec Suite 1.0 (Miotec, Porto Alegre, Brazil) connected to a laptop computer (MSI GP62 2QE Leopard Pro with a Windows 10 operating system) via a USB connection was used to record myoelectric activity. This equipment allows for collecting a maximum of 2000 samples per second.

To capture the electrical potentials (recorded in µV) of the muscles to be studied, the Myotool software incorporated into the electromyograph was used. Of the eight channels that the equipment allows, six were used. The Miograph software (Miotec, Porto Alegre, Brazil) was used for the subsequent visualization and processing of the data.

Adhesive Meditrace Kendall pediatric electrodes—Covidien, Dublin, and Ireland—(Ø = 3 cm) were used, which allowed for more selective recordings due to the spacing between electrodes. These were composed of a white polyethylene foam support with a clasp-shaped adapter and a carbon polymer sensor coated with silver and silver chloride, immersed in a layer of conductive and adhesive hydrogel, to conduct the signal of the muscular electrical activity.

The collection of electromyographic recordings was carried out in a room of the Speech Therapy Clinic of the *Fundació Lluís Alcanyís* of the *Universitat de València*.

### 2.4. Procedure

During the examination, the subjects who were part of the sample remained seated in a chair with knee and hip flexion of 90°, feet on the floor, back of the hands resting on the legs, and with their gaze fixed on a visual reference to the front. They had support on their backs but not on their heads. 

Each participant was asked to swallow to stabilize the larynx, and then the muscles involved in the study were palpated. The neck region corresponding to the suprahyoid, infrahyoid, and sternocleidomastoid muscles was cleaned with gauze soaked in 70° ethyl alcohol (Kern Pharma, Madrid, Spain) before attaching the electrodes. This was intended to decrease the impedance of the skin to better capture the electromyographic signal of the selected muscles and thus provide a better fixation condition for the electrodes [10]. All males who participated in this study had the scanning area completely shaved.

Six electromyography channels (one for each muscle group, namely suprahyoid (SH), infrahyoid (IH), and sternocleidomastoid (ECM), both right (R) and left (L)), and the corresponding 13 disposable electrodes made of hypoallergenic material were used to capture the electrical signal of the muscle: 12 recording electrodes (2 for each channel), separated 1 cm (center to center), and another reference electrode on the olecranon [9]—the most prominent part of the posterior aspect of the elbow—away from the study area to avoid interference.

**Figure 1:**
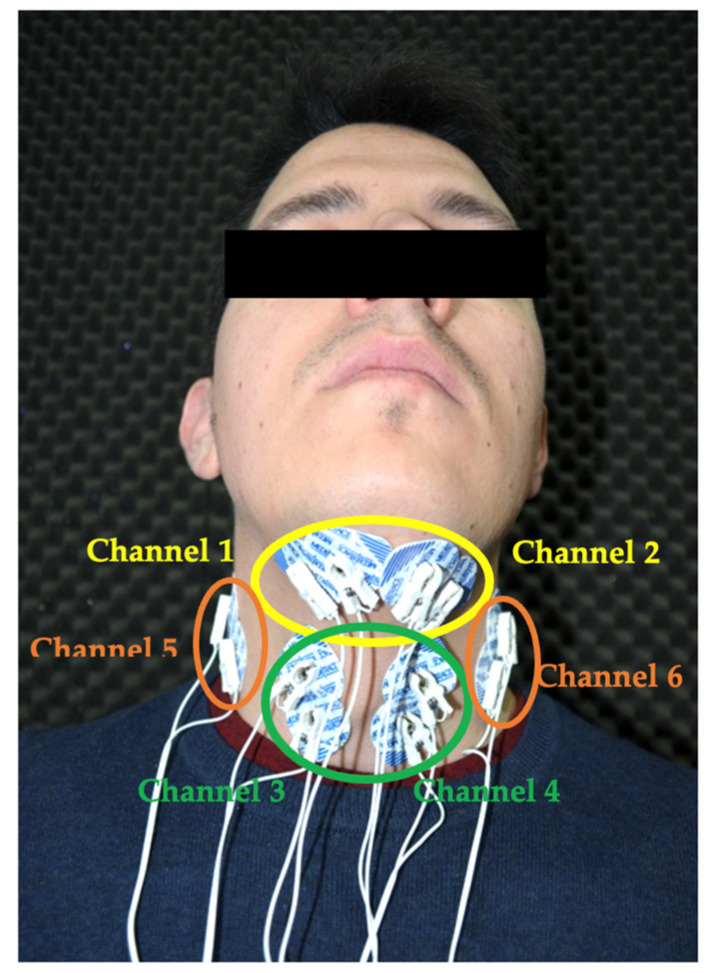
Distribution of SEMG electrodes and channels: Channel 1: R SH; Channel 2: L SH; Channel 3: R IH; Channel 4: L IH; Channel 5: R ECM; Channel 6: L ECM.

**Figure 2:**
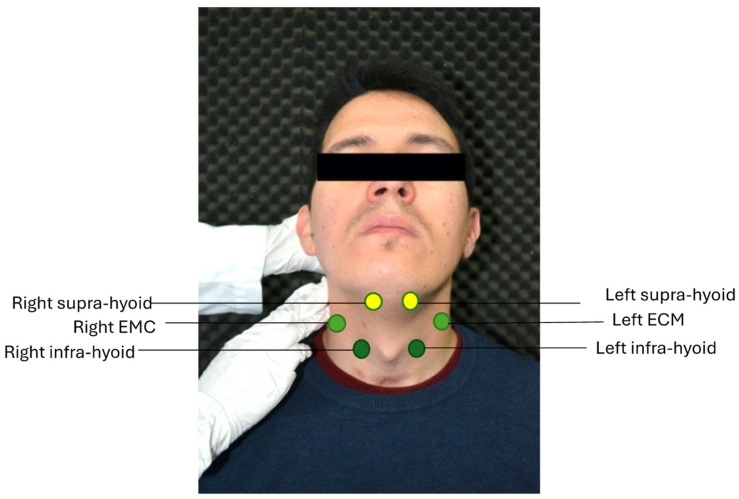
Anatomical positioning of the electrodes.

For the SH muscle group, the electrodes were placed in the submandibular region, longitudinally to the fibers of the mylohyoid and digastric muscles, which are more superficial. For the IH muscle group, they were placed bilaterally and sagittally, 1 cm from the thyroid protuberance. The distance was measured with a digital anthropometric caliper. As for the ECM muscle group, they were positioned in the central part of the muscle in the direction of the clavicle (Figure 1). This is also illustrated anatomically in Figure 2.

Surface electromyographic parameters were analyzed in different vocal tasks (Table 1).

The selection of the SEMG recording period was carried out in coordination with voice recording. External noise was removed from the SEMG signal with Butterworth-type digital filtering. The following data were then obtained (Table 1): peak (µV) (maximum muscle activity), the mean of muscle activation (µV), the minimum value of muscle activation (µV), and standard deviation (µV) and mean frequency (Hz) of the number of times the action potential is repeated per unit of time.

The dynamic normalization method (with dynamic tasks that were neither isotonic nor isometric) was used in this study, as several authors claim that there is strong evidence of reduced inter-subject variability compared to other normalization methods [13].

The “read-aloud” task was chosen as the baseline activity for signal normalization, as it was felt that it could provide much more information than the other isolated tasks.

Electrical activity was also collected during rest or static assessment [4], which was electrically silent and progressively activated in relation to muscle contraction (Table 1).

### 2.5. Statistical Analysis

Data were analyzed using SPSS 26.0 (SPSS Inc., Chicago, IL, USA). Cohen’s kappa test (κ) was used to determine the concordance between external evaluators, and the Kolmogorov–Smirnov test was used to analyze whether the quantitative variables had a normal distribution. A descriptive analysis was performed for each of the sample variables, namely absolute frequency and percentage for qualitative variables and mean and standard deviation for quantitative variables. The Mann–Whitney U test was used to analyze whether the differences observed were statistically significant when dealing with variables that did not have a normal distribution. To analyze quantitative variables that followed a normal distribution, a *t*-test was applied.

## 3. Results

During rest (time of no vocal activity), the myoelectric activity (µV) in women was higher than in men for most of the muscles analyzed, while the frequency of the electrical signal (Hz) was higher in men than in women in all the explored muscles. The results obtained in the suprahyoid (C1 and C2) and sternocleidomastoid (C5 and C6) muscles in both sexes showed statistically significant differences (Table 2).

Regarding the “comfortable /a/ vowel” task, women obtained higher results than men in most of the analyzed variables and explored muscles, except in the channels referring to the infrahyoid muscles (C3 and C4), where men engaged a greater number of motor units and obtained higher results. Statistically significant differences were observed between men and women (Table 3) both in the mean myoelectric activity (µV) and in the mean frequency (Hz) of the electromyographic parameters in each of the recorded channels, except in Channel 3, referring to the right infrahyoid muscle group (*p* = 0.66). The greatest statistically significant differences were observed in the channels of the suprahyoids (C1 and C2). In the left ECM muscle channel (C6), no differences were observed in terms of mean frequency (Hz), while in the right ECM (C5), they were.

There were significant differences in the task “reading aloud” between both sexes (Table 4), in the suprahyoid muscles (C1 and C2) and the peak (µV) and mean (µV) electromyographic parameters (*p* < 0.01).

In the task “ascending glissando with the vowel /i/”, significant differences were observed (Table 5) between men and women in the mean (µV) of the myoelectric activity of the suprahyoid and left infrahyoid and right ECM muscles (*p* < 0.01), as well as the mean electromyographic frequencies (Hz) of the right suprahyoid and left infrahyoid (*p* < 0.01).

In all the previously analyzed tasks, both suprahyoid muscle electrical activity (C1 and C2) and that of the ECM (C5 and C6) were greater (*p* < 0.01) in women, while men presented greater myoelectric activity at the level of infrahyoid muscles (C3 and C4) (*p* < 0.01).

## 4. Discussion

This study aimed to provide information to establish electromyographic normative parameters, typical of voice tasks and where participants present a euphonic voice, not pathological, to help professionals make clinical decisions. For this purpose, the behavior of the extrinsic musculature of the larynx was assessed at the surface electromyographic level during different vocal tasks in men and women with a normal voice. The methodological procedure was based on the results obtained in a recent systematic review of the scientific literature on normal voice [18]. To the best of our knowledge, no studies have been found in Spanish speakers that analyze normal voice in conjunction with surface electromyography.

Balata (2013) [9] studied the electrical activity of extrinsic laryngeal muscles in people with and without dysphonia. His sample consisted of 36 women and 5 men aged between 28 and 57 years. In his study, he used the same equipment as that used in our work (surface electromyograph, adhesive surface electrodes, and software). His analysis included a smaller number of vocal tasks (vowel /ɛ/ at comfortable intensity, vowel /ɛ/ at strong intensity, counting from 20 to 30 at comfortable intensity, and counting from 20 to 30 at strong intensity, together with rest), with three electromyography channels: right and left IH muscles and SH (without specifying the side).

Electromyographic recording depends on electrode placement and application, skin perspiration and temperature, muscle fatigue, contraction speed, muscle bulk, contamination of nearby muscles, subcutaneous fat thickness, or slight variations in the execution of the task [12,13]. Therefore, the signal must be normalized for interpretation and comparison, which is not simple [2]. Lehman and McGill (1999) [12] proposed that electromyographic normalization be performed by means of a reference task, through which the activity values of the electrical signal are expressed as a percentage of the electrical activity of the muscles to be studied during contraction.

The aim is to have a standardized and reliable reference value, by changing the data scale from microvolts to percentages to compare the electrical activity of muscles in a task over time between different muscles and participants [19,20,21].

Research dedicated to the study of the voice in conjunction with SEMG is limited and studies different variables and uses different methodological components such as the muscle groups analyzed, the phonatory tasks recorded, materials, sample size, etc., making it difficult to compare results.

Although SENIAM (surface electromyography for the non-invasive assessment of muscles) is recommended in Europe for the correct placement and location of sensors and is used as the methodology for signal processing, there is currently no specific protocol on the placement of sensors in the extrinsic muscles of the larynx (specifically the suprahyoid (SH) and infrahyoid (IH)) and neck (part of which is the sternocleidomastoid (ECM)). In our case, we decided to place them as previously explained and as shown in Figure 1. Obviously, a consensus standard would be desirable to unify the methodology in this type of research.

In the present study, we chose to study the ECM muscles bilaterally (in addition to the SH and IH) since, according to Behlau and Pontes (1995) [22], these muscles tend to elevate the rib cage constantly, along with the laryngeal elevation and restriction of movements in subjects with vocal pathology. Authors such as Ramos et al. (2018) [23] state that ECMs are muscles in which people with vocal pathology tend to present more pain.

There are other studies using SEMG with vocally normal and dysphonic subjects, although with different objectives. The study by Silvério (1999) [24] analyzed the electrical activity of the ECM and trapezius muscles (upper fibers) in normal and dysphonic individuals. Sapir et al. (2000) [25] evaluated variations in the fundamental frequency together with electrical activity but, in this case, induced by mechanical disturbance of the larynx. Silvério (2002) [26] studied vocal evaluation together with the electrical activity of SH and ECM muscles but in women aged between 20 and 40 years with temporomandibular dysfunction, at rest and phonation. Once again, the variability of the published studies makes comparative analysis difficult.

The recording of the SEMG data required, initially, the precise selection of the period to be studied, taking the time of the voice recordings as a reference. In this way, the exact time window was calculated with SEMG, since it only uses the time variable as a guide for the analysis. To perform both recordings at the same time, the “start” option was selected simultaneously, but on some occasions, the SEMG recording took a few milliseconds longer to start than the vocal recording. For this reason, it was decided to count from the end, stopping the recording again at the same time and ensuring accuracy in data extraction.

The applied digital filtering, Butterworth type [9], attenuates the undesirable frequencies of the signal through two second-order filters, a high-pass filter at 20 Hz that lets through the frequencies above that frequency and a low-pass filter at 450 Hz, letting through the frequencies below that frequency [27], which were configured as cutoff frequencies to define the upper and lower filtering limits since the highest-frequency components contained in the SEMG signals are below 400–500 Hz [7].

The dynamic normalization method (with dynamic tasks that were neither isotonic nor isometric) was used in this study, as it has been revealed that there is strong evidence of reduced inter-subject variability compared to other normalization methods [13,20]. The “read-aloud” task was chosen as the reference activity for signal normalization, as it was considered to be one that could provide more information than the other isolated tasks. Electrical activity during rest (statically) was also recorded, which is electrically silent and allows for the assessment of the muscle without load [28]. In a general way, it can be seen how “reading aloud” is the task that recruits more motor units than the others, taking “rest” or “no muscle activity” as a reference.

During the task “upward glissando with the vowel /i/”, the mean myoelectric activity was slightly higher in the infrahyoid and ECM muscles at low frequencies, while the mean myoelectric activity of the suprahyoids was slightly higher at high frequencies. A plausible explanation for the higher amplitude of the ECM electrical signal at low frequencies may be due to the onset of inhalation (high/clavicular breathing may have occurred at most phonatory onsets) or a very rapid inhalation at the time of first uttering a low frequency [29]. Another reasonable, although unconfirmed, explanation would be the possible involvement of ECMs in an attempt to lower the larynx at lower pitches. The higher amplitude at high frequencies in the supra-hyoid muscles is due to the laryngeal rise that occurs during higher-pitched vocal emissions, due to the increased activity of the cricothyroid muscle responsible for the progressive elevation of the vocal ligament [30,31].

In our work, in general (since several SEMG channels were analyzed in different vocal tasks), women presented a higher mean myoelectric activity (µV) at the SH and ECM muscle levels than men, while the mean frequency of the electrical signal (Hz) was higher in men than in women. This may be due to the fact that the higher the amplitude of the SEMG (µV) (i.e., the higher the recruitment of motor units), the lower the conduction velocity of muscle action potentials (i.e., the lower the SEMG frequencies (Hz)) [32]. The changes that may occur at the level of the amplitude and frequency of the SEMG signal are related to constant changes in the performance of force, the length of the muscle fibers, the placement/position of the surface electrodes, and the active muscle fibers during the different tasks [33].

An important aspect to take into account is the variety of vocal tasks that were studied in the present study. It is important to consider that continuous speech is a much more complex task than sustained vocal emission because, in the former, many more orofacial structures are activated.

The results obtained regarding the mean muscle electrical activity of the SH and ECM muscles, both in men and women, presented statistically significant differences in our work, aspects that cannot be compared due to the lack of similar studies. Research by Silvério (1999) [24] and Balata (2013) [9] compared dysphonic and euphonic subjects and did not analyze differences between sexes. The analysis of SEMG results revealed a large interindividual variation, even in subjects performing the same task [34,35].

The results of the present study, performed on a population without pathology, show differences by gender, but these obviously cannot be extrapolated to populations with dysphonia. On the other hand, the scarcity of evidence in this field does not allow us to do so either.

SEMG is not widely used in clinical speech therapy practice (recognition of dysfunctions) or the control of therapy; these cases involve procedures for recording information that is not uniform, which implies the translation of the analyses performed [4]. It is evident that without a methodological standardization that minimizes individual variations [24], it is not possible to adequately broaden the spectrum of knowledge in this field, since the capacity for comparison with other studies is limited.

## 5. Conclusions

Within the characteristics and limitations of this study, the following conclusions can be drawn:

SEMG provides valid and objective data on the extrinsic muscular activity of the larynx during phonation, but this tool cannot precisely define such markers since the electrophysiological assessment of phonation is subject to many individual variables. On the other hand, we wish to highlight the potential of surface electromyography as biofeedback and the benefits that its use can bring from a clinical point of view.

The dynamic tasks related to connected speech best describe the myoelectric behavior of the extrinsic structures of the larynx involved in the phonatory process. The mean myoelectric activity of the suprahyoid musculature and that of the sternocleidomastoid muscles was higher in women, whereas the mean myoelectric activity of the infrahyoid musculature was higher in men.

Therefore, the proposed hypotheses are accepted. The behavior of the SEMG signals was analyzed, and differences between sexes were observed for the studied population. However, it is necessary to establish a system of standardization of the scans and conduct further research to make the data comparable with each other and with other populations.

## Figures and Tables

**Table 1:** Surface electromyographic parameters studied in selected vocal tasks.

Vocal Task	Electromyographic Surface Parameters Studied
Reading aloud (SEMG signal normalization task)	Peak (µV)
Vowel /a/ at comfortable frequency and intensity	Mean (µV)
	Minimum (µV)
Upward *glissando* with the vowel /i/ (low and high part)	Standard deviation (µV)
Rest (time of no vocal activity)	Average frequency (Hz)

Peak (µV): numerical result of the maximum value of electrical activity recorded in the interval analyzed; mean (µV): average value of electrical activity recorded in the interval selected for analysis; minimum (µV): numerical result of the minimum value of electrical activity recorded in the interval analyzed; standard deviation (µV): difference found from the mean value to the extremes of maximum and minimum electrical activity recorded in the interval selected for analysis; mean frequency (Hz) [17].

**Table 2:** Resting muscle electrical activity in men and women.

		Men	Women		Men	Women	
		µV	µV	*p*	Hz	Hz	*p*
C1	Mean	6.67	9.11	<0.01	345.98	311.95	<0.01
	SD	4.27	6.66		59.14	54.79	
C2	Mean	7.50	7.77	<0.05	286.00	266.12	<0.01
	SD	6.08	3.99		41.09	38.81	
C3	Mean	6.05	6.16	0.53	339.03	332.16	0.28
	SD	2.57	2.72		33.18	40.16	
C4	Mean	5.48	5.14	0.90	362.52	358.68	0.19
	SD	3.76	2.39		67.33	37.24	
C5	Mean	12.79	20.40	<0.05	245.20	212.61	<0.01
	SD	10.31	18.41		52.98	54.82	
C6	Mean	6.71	7.03	<0.05	331.22	303.57	<0.01
	SD	6.59	4.49		48.18	51.50	

C1: right suprahyoid channel 1; C2: left suprahyoid channel 2; C3: right infrahyoid channel 3; C4: left infrahyoid channel 4; C5: right ECM channel 5; C6: left ECM channel 6; C7: right ECM channel 6; C8: right ECM channel 6; C9: right ECM channel 6. SD: standard deviation.

**Table 3:** Resting muscle electrical activity in the task “vowel /a/ at comfortable frequency and intensity” in men and women.

		Men	Women		Men	Women	
	µV		µV	*p*	Hz	Hz	*p*
C1	Mean	10.11	13.49	<0.01	296.17	275.61	<0.01
	SD	1.86	2.52		41.87	39.16	
C2	Mean	10.56	12.59	<0.01	269.65	253.68	<0.05
	SD	1.94	2.67		38.37	30.69	
C3	Mean	11.91	11.35	0.66	264.13	268.93	0.56
	SD	3.08	2.45		44.51	40.12	
C4	Mean	12.98	10.01	<0.05	265.44	271.87	0.38
	SD	3.56	2.14		4911	38.74	
C5	Mean	12.75	21.47	<0.01	252.79	236.37	<0.01
	SD	1.10	1.59		45.20	57.72	
C6	Mean	8.41	9.99	<0.01	297.31	278.33	0.07
	SD	0.93	1.64		56.99	55.97	

C1: right supra-hyoid channel 1; C2: left supra-hyoid channel 2; C3: right infra-hyoid channel 3; C4: left infra-hyoid channel 4; C5: right ECM channel 5; C6: left ECM channel 6.

**Table 4:** Muscle electrical activity in the task “reading aloud” in men and women.

		Men	Women		Men	Women	
	Mean (µV)		Mean (µV)	*p*	Mean Frequency (Hz)	Mean Frequency (Hz)	*p*
C1	Mean	20.57	29.36	<0.01	229.91	219.99	<0.05
	SD	8.17	10.70		23.03	22.18	
C2	Mean	22.81	29.42	<0.01	219.58	213.80	0.12
	SD	9.72	11.46		19.99	17.43	
C3	Mean	22.72	21.06	0.19	209.85	216.02	0.10
	SD	9.45	7.92		31.18	33.53	
C4	Mean	25.46	18.87	<0.01	202.61	220.49	<0.01
	SD	11.13	7.14		27.51	25.16	
C5	Mean	15.84	21.09	<0.05	237.78	228.84	0.12
	SD	2.84	2.79		30.07	24.47	
C6	Mean	11.15	11.09	0.17	269.53	257.43	0.09
	SD	3.57	2.92		48.32	33.04	

C1: right supra-hyoid channel 1; C2: left supra-hyoid channel 2; C3: right infra-hyoid channel 3; C4: left infra-hyoid channel 4; C5: right ECM channel 5; C6: left ECM channel 6.

**Table 5:** Muscle electrical activity in the task “upward glissando with /i/” in men and women.

		Men	Women		Men	Women	
	Mean (µV)		Mean (µV)	*p*	Mean Frequency (Hz)	Mean Frequency (Hz)	*p*
C1	Mean	11.34	16.35	<0.01	280.08	254.95	<0.01
	SD	2.04	2.89		41.73	36.54	
C2	Mean	12.80	15.30	<0.01	251.92	239.84	<0.05
	SD	2.50	2.82		36.13	32.66	
C3	Mean	24.34	16.12	<0.01	232.93	249.36	<0.01
	SD	8.34	4.01		46.11	41.77	
C4	Mean	25.92	13.59	<0.01	236.10	263.03	<0.01
	SD	8.60	3.49		47.93	47.69	
C5	Mean	12.88	2096	<0.01	239.73	220.68	<0.01
	SD	1.33	1.33		38.84	42.89	
C6	Mean	7.96	9.37	<0.01	299.98	279.11	<0.01
	SD	0.90	1.41		52.59	41.49	

C1: right supra-hyoid channel 1; C2: left supra-hyoid channel 2; C3: right infra-hyoid channel 3; C4: left infra-hyoid channel 4; C5: right ECM channel 5; C6: left ECM channel 6.

## Data Availability

Not applied.
